# The unmet global burden of COPD

**DOI:** 10.1017/gheg.2018.1

**Published:** 2018-04-06

**Authors:** S.A. Quaderi, J.R. Hurst

**Affiliations:** UCL Respiratory, University College London, London, UK

**Keywords:** Chronic obstructive pulmonary disease, smoking, air pollution, Low and middle income countries

Chronic respiratory diseases receive little attention and funding in comparison with other major causes of global morbidity and mortality [1]. Chronic obstructive pulmonary disease (COPD) is a major public health problem. COPD is the end result of a susceptible lung being exposed to sufficient environmental stimulus. Caused principally by tobacco smoking and household air pollution (HAP), COPD is a silent killer in low- and middle-income countries (LMICs): an estimated 328 million people have COPD worldwide [2], and in 15 years, COPD is expected to become the leading cause of death [3].

The relentless decline in lung function that characterises COPD is associated with progressive symptoms and functional impairment, with susceptibility to respiratory infections called ‘exacerbations’. Exacerbations are responsible for much of the morbidity and mortality. COPD has a significant impact on quality of life for those living with the condition, and on local economies for those affected, those caring for the affected and health services. A population literally struggling for breath is, in consequence, unproductive. The majority of cases of chronic lung disease are preventable. Exposure reduction initiatives must focus on tobacco control, and cook-stove interventions: either cleaner fuel (ideally), or better ventilation (at the least). Awareness campaigns and health programmes have the potential to revolutionise the diagnosis and management of COPD and COPD exacerbations, improving quality of life and health service cost and burden. LMICs face unique challenges in managing COPD, including sub-optimal and diverse primary care systems which present challenges with diagnosis and management, especially during exacerbations. A better understanding of how to prevent, diagnose and manage COPD in both rural and urban settings would make a real difference in countries of need.

Two important aspects to consider when addressing the global economic burden of COPD are that of underdiagnosis and comorbidities [4]. Firstly, COPD remains underdiagnosed in many jurisdictions [5]. Studies included in reviews focusing on the global economic burden of COPD are all based on diagnosed COPD, and a simple multiplication of these values by the number of COPD patients to calculate the overall economic burden of COPD will underestimate the contribution of undiagnosed COPD [5]. Secondly, COPD is known to be associated with a significant number of comorbid conditions, and estimating costs that are directly attributable to COPD fails to consider the burden of such comorbidities [4]. Adjusting for comorbidities by calculating excess costs with an appropriate comparison group can provide a better opportunity, but even this results in an underestimation of the costs of the comorbidities [6–8].

## Global COPD statistics

- More than 90% of COPD-related deaths occur in LMICs [3].
- According to the Global Burden of Disease (GBD), COPD is already the third leading cause of death worldwide, something that WHO had not predicted to occur until 2030 [9].
- The economic impact of COPD among LMICs is expected to increase to £1.7 trillion by 2030 [10].
- In 15 years, COPD is expected to become the leading cause of death worldwide [3].

## Air pollution and HAP

Air pollution is the biggest *environmental* cause of death worldwide, with HAP accounting for about 3.5–4 million deaths every year [11]. Extensive literature supports a causal association between HAP and chronic lung diseases [12], respiratory infections and respiratory tract cancers. One-third of the world's population, some three billion people use fuel derived from organic material (biomass) or solid fuel including coal, wood and charcoal as an energy source to heat and light their homes, and to cook. Respiratory morbidity relates to products of incomplete combustion such as carbon monoxide, and to particulate matter (PM).

PM include both organic and inorganic particles, and represents the sum of all solid and liquid particles suspended in the air, many of which are hazardous. PM_10_ is the most widely used indicator of the health hazard of indoor air pollution. The EU and the US Environmental Protection Agency have set standards for annual mean PM_10_ levels in outdoor air at 40  and 50 µg/m^3^, respectively [13]. When burning solid fuels, peak levels of PM_10_ in biomass-using homes can be as high as 10 000 µg/m^3^, 200 times more than the standard in high-income countries. PM_2.5_ are finer particles which penetrate deep into the lung and have the greatest health-damaging potential. Pollutants are particularly damaging and of concern to children growing up in homes with HAP, the effect on the developing lung results in lung function that does not reach maximum potential.

## Household air pollution statistics

- Air pollution is the biggest *environmental* cause of death worldwide [14].
- Three billion people worldwide are exposed to toxic amounts of HAP every day [11].
- HAP accounts for up to four million deaths annually [11].

## Smoking

Tobacco is a legal drug which is currently responsible for the deaths of an estimated six million people across the world each year, with many of these deaths occurring prematurely [15]. Tobacco smoking is associated with morbidity and mortality from non-communicable respiratory diseases (NCDs), including about 600 000 people who are estimated to die every year from the effects of second-hand smoke [15].

The poor tend to smoke the most. Globally, 84% of smokers live in developing and transitional economy countries [16]. Tobacco smoke potentiates the detrimental effects of biomass smoke exposure. The WHO stated that in 2015, over 1.1 billion people smoked tobacco, males smoked tobacco more than females, and although it is declining worldwide and in many countries, the prevalence of tobacco smoking appears to be increasing in the Eastern Mediterranean and Africa [17].

## Tobacco and smoking statistics

- Due to the incomplete combustion of formaldehyde and DEET, one mosquito coil burning for 8 h releases the same amount of PM_2.5_ as 100 cigarettes [18].
- A 1 h hookah session with shisha tobacco is equivalent to smoking over 100 cigarettes [19].
- Those who have never smoked tobacco **can** still get COPD – think ‘biomass COPD’.

## COPD: under-recognition and inequity

There is a need for governments, policy makers and international organizations to consider strengthening collaborations to address COPD. TB, HIV/AIDS and malaria all compete for headlines and funds; COPD is rarely the headline. There is global under-recognition of COPD. This needs to change and we welcome recent initiatives highlighting unmet needs in NCDs. The United Nations (UN) declaration of NCDs, and the World Health Assembly in 2012, endorsed a new health goal (the ‘25 by 25 goal’), which focuses on the reduction of premature deaths from COPD and other NCDs by 25% by the year 2025 [20]. Many NCDs occur together in the context of multi-morbidity, yet despite this initiative, COPD remains a growing but neglected global epidemic. It is under-recognised, under-diagnosed and under-treated resulting in millions of people continuing to suffer from this preventable and treatable condition.

The lower an individual's socio-economic position, the higher their risk of poor health: women and children living in severe poverty have the greatest exposures to HAP [21]. In the poorest countries, cooking with solid fuels can be the equivalent of smoking two packs of cigarettes a day [22]. A 1-year old would have accumulated a two pack year smoking history having never seen tobacco. Inaction to mitigate COPD therefore exacerbates health inequalities.

Climbing the ‘energy ladder’ occurs gradually as most LMIC households use a combination of fuels. The poorest, at the bottom of the ladder, use crop waste or dung which is the most harmful when undergoing incomplete combustion. Those at the top of the ladder use electricity or natural gas. Increasing prosperity and development has a direct positive correlation with increasing use of cleaner and more efficient fuels for cooking [23].

The unmet global burden of COPD is a silent killer in LMICs. In conclusion, we suggest that given the high and rising global burden of COPD, a revolution in the diagnosis and management of COPD and exacerbations of COPD in LMICs must be an urgent priority.

## Summary

- An estimated 328 million people have COPD worldwide [3].
- In 15 years, COPD is expected to become the leading cause of death worldwide [3].
- Three billion people worldwide are exposed to toxic amounts of HAP every day and HAP accounts for 3.5–4 million deaths annually [11].
- Those who have never smoked tobacco **can** still get COPD – think ‘biomass COPD’.
